# A Pragmatic Randomised, Controlled Trial of Intensive Care follow up programmes in improving Longer-term outcomes from critical illness. The PRACTICAL study

**DOI:** 10.1186/1472-6963-7-116

**Published:** 2007-07-23

**Authors:** Brian H Cuthbertson, Janice Rattray, Marie Johnston, J Anthony Wildsmith, Edward Wilson, Rodolfo Hernendez, Craig Ramsey, Alastair M Hull, John Norrie, Marion Campbell

**Affiliations:** 1 Health Service Research Unit, Health Sciences Building, University of Aberdeen, Foresterhill, Aberdeen, AB25 2ZD, UK; 2 School of Nursing and Midwifery, University of Dundee, Ninewells Hospital, Dundee, DD1 9SY, UK; 3 Department of Anaesthesia, University of Dundee, Ninewells Hospital Dundee, DD1 9SY, UK; 4 Department of Anaesthetics, Ninewells Hospital Dundee, DD1 9SY, UK; 5 Murray Royal Hospital, Perth PH2 7BH, UK

## Abstract

**Background:**

A number of intensive care (ICU) patients experience significant problems with physical, psychological, and social functioning for some time after discharge from ICU. These problems have implications not just for patients, but impose a continuing financial burden for the National Health Service. To support recovery, a number of hospitals across the UK have developed Intensive Care follow-up clinics. However, there is a lack of evidence base to support these, and this study aims to test the hypothesis that intensive care follow up programmes are effective and cost-effective at improving physical and psychological quality of life in the year after intensive care discharge.

**Methods/Design:**

This is a multi-centre, pragmatic, randomised controlled trial. Patients (n = 270) will be recruited prior to hospital discharge from three intensive care units in the UK, and randomised to one of two groups. The control group will receive standard in-hospital follow-up and the intervention group will participate in an ICU follow-up programme with clinic appointments 2–3 and 9 months after ICU discharge.

The primary outcome measure is Health-related Quality of Life (HRQoL) 12 months after ICU discharge as measured by the Short Form-36. Secondary measures include: HRQoL at six months; Quality-adjusted life years using EQ-5D; posttraumatic psychopathology as measured by Davidson Trauma Scale; and anxiety and depression using the Hospital Anxiety and Depression Scale at both six and twelve months after ICU discharge. Contacts with health services in the twelve months after ICU discharge will be measured as part of the economic analysis.

**Discussion:**

The provision of intensive care follow-up clinics within the UK has developed in an ad hoc manner, is inconsistent in both the number of hospitals offering such a service or in the type of service offered. This study provides the opportunity to evaluate such services both in terms of patient benefit and cost-effectiveness. The results of this study therefore will inform clinical practice and policy with regard to the appropriate development of such services aimed at improving outcomes after intensive care.

**Trial Registration:**

ISRCTN24294750.

## Background

Over 100,000 patients are admitted to intensive care units in the United Kingdom (UK) per year [1]. Of these over 40,000 are dead within one year of admission [2]. Over the five years after an intensive care unit (ICU) admission these patients also have an excess risk of death when compared to an age and sex matched population [3]. Apart from this excess mortality, it is now becoming clear that intensive care patients continue to experience both physical (e.g. neuropathy, reduced mobility, and breathlessness) [4,5], and psychological problems (e.g. anxiety, depression and posttraumatic stress) [6-9] for some time after discharge from ICU. Approximately two thirds of ICU survivors will experience significant problems with physical and psychological health and social functioning, and with 13% of patients experiencing severe limitations in every day life [10,11]. Studies assessing Health-Related Quality of Life (HRQoL) after intensive care suggest that this improves over time [12,13], but is worse than before admission to ICU [14,15], and worse than general population norms [13,16,17]. Reported prevalence of anxiety and depressive problems in this patient group ranges from 12% to 43% (for anxiety) [9,16], and 10% to 30% (for depression) [9,16]. Reports also suggest that 14% to 27% of intensive care patients may develop a posttraumatic stress reaction [6,9] that may endure for a number of years [7]. Patients' perceptions of the intensive care experience itself are associated with subsequent distress, and patients' subjective reports frequently include the presence of 'odd perceptual experiences' [18], or 'nightmares' and 'hallucinations' [19] which seem real and distressing for them at the time. The reported frequency of these experiences ranges from 7% to 73% [20,21]. These continuing problems have implications not just for patients, but impose a continuing financial burden for the National Health Service (NHS) in terms of primary and secondary health care costs.

Eighty hospitals across the UK have now developed Intensive Care follow-up clinics in an attempt to improve outcomes after ICU discharge [22]. This is despite a lack of an evidence base for this intervention, although the Outreach Forum of the Department of Health has issued guidelines for the delivery of ICU follow-up services based on expert opinion [11]. Despite these guidelines, it is clear that the nature of these clinics varies between centres [22]. The clinics aim to support recovery, are often multi-disciplinary in nature and offer patients the opportunity to discuss issues related to their intensive care experience over two or three appointments in the year after ICU discharge [22]. One of the other purported benefits of these clinics is that ICU practitioners gain valuable information about psychological and physical problems faced by patients that can then be used to inform the management of future patients [4,5]. The NHS is spending a large amount of clinical resource on these clinics and this is likely to continue. Because there are only 80 ICU follow-up clinics in the UK currently (30% of ICUs), with a large variety of models and funding structures, there exists a window of opportunity to evaluate this intervention before the introduction of such clinics becomes more widespread.

## Aims and objectives of the study

This study aims to test the hypothesis that intensive care follow up programmes are effective and cost-effective at improving physical and psychological quality of life in the year after intensive care discharge. The study will investigate also these outcomes in patient subgroups classified according to illness severity, intensive care experience, anxiety and depression and ICU length of stay. In addition, the study will establish the cost-effectiveness of such clinics.

## Methods/Design

This is a multi-centre, pragmatic, randomised controlled trial. Patients will be randomised to one of two intervention groups after ICU discharge but prior to discharge from hospital.

### Ethics Approval

Ethics approval was granted from the Fife and Forth Valley Local Research Ethics Committee (Ethics Code: 06/S0501/26).

### Participants

Participants meeting the inclusion criteria will be recruited from three intensive care units in the UK (2 teaching hospitals and 1 District General hospital).

#### Inclusion criteria

Patients receiving level three dependency care (ICU) at any time during their hospital stay and who survive until the time of hospital discharge [23]. There is no evidence to guide which patients should be offered this service, thus we have chosen to include all patients irrespective of ICU length of stay.

#### Exclusion criteria

Patients will be excluded if they are: aged under 18 years; not expected to survive to leave hospital; unable to complete questionnaires; unable to attend clinics, or if they are unable or unwilling to consent.

### Outcome measures

#### Primary measures

HR-QOL 12 months after ICU discharge as measured by the physical and mental component scores of the Short Form-36 (SF-36) [24,25]. This is a comprehensive, generic 36-item questionnaire [26,27], well accepted by different patient groups, is reliable [27,28], valid [26,29], shows sensitivity to change over time [30,31], and takes minutes to complete [32]. The SF-36 has been widely used in this patient population [2].

#### Secondary measures

HR-QOL will be assessed at six months after ICU discharge. Quality-adjusted life years (QALYs) using EQ-5D [33]; incidence and severity of posttraumatic psychopathology as measured by Davidson Trauma Scale (DTS); anxiety and depression using Hospital Anxiety and Depression Scale (HADS) will be assessed at both six and twelve months. Contacts with health services measured as part of the economic analysis; patient satisfaction will be assessed at twelve months using a patient satisfaction survey; primary and secondary health care costs in the year after hospital discharge, and mortality in the twelve months after ICU discharge. All non-clinic interventions will be monitored and documented. These data will be collected from hospital case notes review and patient questionnaires.

The EQ-5D assesses five attributes of quality of life, namely mobility, self-care, usual activity, pain/discomfort, and anxiety and depression [2]. An overall single score is obtained which can then be converted into a utility score. The DTS [34] will assess posttraumatic psychopathology and the HADS [35], anxiety and depression. Both the DTS and HADS have been validated in this patient population [6,9,16,19]. The DTS has been used widely in the initial assessment of patients at risk of posttraumatic stress disorder (PTSD). It measures all core symptoms of PTSD i.e. intrusion, avoidance and hyperarousal and has a 'cut-off' score of 27 or more that indicates significant posttraumatic psychopathology whilst a score of 40 or more indicates a likely diagnosis of PTSD [34]. The HADS questionnaire contains 14 statements and scoring results in scales of 0–21 for anxiety and depression respectively. Scores of 8–10 indicate the possibility of anxiety or depression, and 11 and above indicate that these are likely to be present.

The Intensive Care Experience Questionnaire (ICE) will be used to assess patients' perceptions of their experience [8]. This measure assesses four aspects of such an experience – 'awareness of surroundings', 'frightening experiences', 'recall of experiences', and 'satisfaction with care'. Previous work has demonstrated its association with both short and long-term psychological outcome [8], and we have used the median value from the 'frightening experiences' aspect as the score for the randomisation procedure.

### Intervention Group

These patients will be randomised to the ICU follow up programme and attend clinic appointments at 2 – 3 months and 9 months after hospital discharge. The design and timing of these clinics has been determined by the guidelines on ICU follow up from the Outreach Forum [11] and by the information collected from our survey of current practice. Clinics will be primarily nurse-led with support from an intensive care doctor. This doctor will be available to consult with the patient (at the nurse's request) to discuss medical aspects of the patient's original ICU stay or ongoing treatment issues relating to ICU management.

Evidence suggests that a self-directed rehabilitation programme may improve outcome after ICU [19]. Therefore immediately after enrolment patients will be offered a self-directed rehabilitation programme to follow at home. This will be individualised to each patient after a discussion between the clinic nurse and a physiotherapist involved in the patient's hospital care.

Prior to the clinic appointments the ICU follow-up nurse will review the patient's case notes and clinical audit database to highlight any specific issues which might have arisen between discharge and the clinic appointment. The clinic consultation will be structured using a proforma which has been developed in centres with experience of following up these patients. Patients and relatives will have the opportunity to discuss their ICU experiences and this will be informed by responses to specific items included in the proforma and also by a free ranging discussion led by the patients and their families.

Physical recovery will be assessed at the clinic using a structured questionnaire currently used by one of the existing centres. This is divided into two sections with one asking about both general health and specific problems or changes since being in intensive care. These reflect our current understanding of such problems, [4,5] for example hoarseness, breathlessness, eating difficulties, joint stiffness, parasthesia, concentration and memory difficulties. Where indicated patients may be referred to specialist services e.g. physiotherapy. Psychological recovery will be assessed using the standardised screening measures, and patients scoring above the 'cut-point' scores will be referred to a mental health professional (such as a clinical psychologist, psychiatrist or mental health nurse) for review.

Current drug therapy will be reviewed. It is common for drugs started during the ICU stay to be continued unnecessarily long after hospital discharge. The clinic nurse will review current medications and, in liaison with the ICU doctor, will consider the appropriateness of continuing these therapies.

Patients will be offered an ICU visit if appropriate at an other time. The presence of altered memories and delusional ideation are related to severe psychological morbidity after ICU discharge. A return visit to ICU will only be offered if the patient has no psychological problems on screening (or after assessment by mental health professional if caseness was established on screening). For certain patients a visit to the ICU is recognised to help to develop greater understanding of their memories and delusions, and may aid recovery.

After each clinic visit, a review letter explaining the patient's progress will be sent to the general practitioner (GP). For good clinical practice the patient's GP will be informed of the patient's status and any referrals required as a result of the clinic visit. All interventions will use standard NHS processes, which reflect the pragmatic nature of this study.

The interventions are clearly specified and, wherever possible, chosen to replicate the standard processes of the NHS in the UK to allow the study intervention to be reproduced accurately in all study centres. However, to ensure that trial patients always receive the appropriate trial interventions we have also organised direct, assured and named pathways for referral to specialist services.

We have chosen to have three trial centres with one study nurse carrying out the intervention in each centre. These nurses will be trained together by nurses who currently carry out ICU follow up programmes to ensure a high level of standardisation; their application of the protocol will be monitored.

### Standard care

In line with standard clinical practice in the UK patients allocated to the standard care group will have no intensive care follow-up after hospital discharge. Patients will be followed-up for the trial outcome measures and end points only over the first year after hospital discharge. In line with good clinical practice, if there are concerns about the well being of these patients at trial follow-up, a GP letter will be generated.

### Procedure

#### Trial recruitment and baseline measurements

Patients will be approached in the period after ICU discharge and before hospital discharge, when their condition has been identified as stable and they are able to give informed consent. The local trial nurse will explain the trial and allow time for the patients to read an information sheet before seeking written informed consent. Patients will have baseline measurements recorded before allocation to their study group. Clinically-gathered data on patient demographics, ICU diagnosis, ICU stay, severity of illness using the Acute Physiology, Age and Chronic Health Evaluation (APACHE II) system [36], APACHE II co-morbidity score, ICU interventions and sedative use will be extracted from clinical audit data which is collected routinely during ICU stay in all units and available at time of enrolment. Also at baseline we will measure SF-36, EQ-5D, ICE score and HADS score before group allocation.

#### Allocation of participants to trial groups

After recruitment the patients will be randomised using the existing central automated computerised telephone randomisation service within the Centre for Healthcare Randomised Trials (CHaRT) in the Health Services Research Unit in the University of Aberdeen. Allocation will incorporate randomisation at baseline according to age, sex, HADS score, APACHE II, ICE questionnaire score and trial centre

#### Methods for protecting against other sources of bias

Due to the nature of the trial intervention, the nurses and doctors performing trial intervention cannot be blind to the trial group allocation. The researchers who measure trial end points and who will analyse the data will be blinded to the trial groups. To reduce bias, the principal outcome measures are questionnaires completed by participants at a time equivalent to three months after each clinic assessment. The analyses will be based on the 'intention to treat' principle including patients who cannot attend clinics due to psychiatric treatment.

#### Frequency and duration of follow up

Patients will be followed-up at six and twelve months for the patient-assessed measures of outcome. Postal questionnaires, rather than in-person assessment, have been chosen to avoid the six months outcome measurement acting as a trial intervention. This will also help to standardise the method of data collection.

#### Sample size

Previous studies performed in Aberdeen have shown a SF-36 mean physical component score (PCS) of 35 (S.D. 14) in ICU patients one year after ICU discharge (50 being the population norm). We have powered this study to detect a difference of 5 PCS points (0.36 SDs) increase in the clinic group compared to the standard care group (i.e. 35 to 40). For an unadjusted analysis, 123 patients per group would be required to detect this difference with 80% power and α of 0.05. However, data from previous studies in Aberdeen [2] indicate that the correlation between baseline, 3 month and 12 month SF-36 scores were at least 0.6. Such high correlations imply that the sample size could be reduced by 36% and the study would still detect a 5-point difference. Taking a more conservative estimate of 30% reduction in sample size, we would need 86 patients per group (172 total) to detect this difference with an 80% power at an α of 0.05. In practice, the power should be greater than 80% once the randomisation variables have been included in the statistical modelling. In our previous work we have found that 20% of patients who are discharged alive from hospital are lost to follow up/do not return questionnaires over the first year [2,6,8]. We will attempt to minimise this form of trial loss by keeping records of patients' telephone contact details as well as secondary contact names and addresses. All patients will be contacted by phone if they fail to return questionnaires and asked if they want to continue with the study. Nevertheless, we are assuming a 20% loss to follow-up and 20% mortality at 12 months; thus, the total number of patients to be recruited will be inflated to 135 per group or 270 in total.

#### Planned analyses

The outcomes will be compared between groups using generalised linear models which adjust for the randomisation factors. The model used will reflect the type of outcome data. For the primary outcome statistical significance will be at the 5% level (2P < 0.05) and 95% confidence intervals will be derived. *A priori *subgroup analysis will be undertaken for severity of illness (APACHE II), APACHE II co-morbidity, ICE score [8] and ICU length of stay. All subgroup analyses will seek stricter levels of significance (2P < 0.01). Data will be analysed using SPSS software.

#### Economic issues

A formal economic evaluation is an integral part of this project. Cost per participant for each arm of the trial will be calculated. An estimate of primary and secondary care resource utilisation per patient will be made using patient questionnaires and review of hospital notes over the first year after hospital discharge. Unit costs/prices will be obtained using published estimates for health care services and/or interventions as well as study specific estimates [37-39]. QALYs will be calculated using the area under the curve method. For this, EQ-5D questionnaire responses will be valued using UK population tariffs [33]. The estimation of QALYs will take account of the mortality of study participants. Participants who die within the follow-up period will be assigned a zero utility weight from their death to the end of the follow-up. QALYs before death will be calculated using linear extrapolation between QALY scores. Point estimates for mean costs and mean QALYs will be derived for treatment and control groups to obtain Incremental Cost per QALY gained. Measures of variance of outcomes are likely to involve bootstrapping estimates for costs and QALYs. Results will be presented using Cost Effectiveness Acceptability Curves (CEACs). Deterministic and probabilistic sensitivity analysis will be developed to address different types of uncertainties within the economic evaluation such as differences in death rate. An element of this sensitivity analysis will be to explore the impact of extrapolating to a longer time than that afforded by the trial. This will be informed by previous research in which we have been involved [2,6,8], and will use a variety of modelling techniques. Subgroup analysis will follow the subgroup definitions within the trial.

## Conclusion

The provision of intensive care follow-up clinics within the UK has developed in an ad hoc manner, is inconsistent in both the number of hospitals offering such a service or in the type of service offered. This study provides the opportunity to evaluate such services both in terms of patient benefit and cost-effectiveness. The results of this study therefore will inform clinical practice and policy with regard to the appropriate development of such services aimed at improving outcomes after intensive care.

## Abbreviations

UK: United Kingdom

ICU: Intensive Care Unit

HRQoL: Health-related Quality of Life

NHS: National Health Service

SF-36: Short-form 36

QALYs: Quality-adjusted life years

DTS: Davidson Trauma Scale

HADS: Hospital Anxiety and Depression Scale

PTSD: Posttraumatic Stress Disorder.

ICE: Intensive Care Experience Questionnaire

## Competing interests

The author(s) declare that they have no competing interests.

## Authors' contributions

All authors contributed to the writing of the study protocol, are grant holders and approved the final version. The study was initiated by BH, JR, MJ, and JAW. MJ, CR, JN, MC, RH provided expertise in clinical trials, statistical and economic evaluation. BH, JR, EW and AH provided intensive care and posttraumatic stress expertise.

## Pre-publication history

The pre-publication history for this paper can be accessed here:


